# Unveiling the immunopathology of stroke: a comprehensive view on brain-immune interaction

**DOI:** 10.1007/s00281-023-00995-3

**Published:** 2023-06-19

**Authors:** Tim Magnus, Arthur Liesz

**Affiliations:** 1 Department of Neurology, University Medical Center Hamburg-Eppendorf, 20246 Hamburg, Germany; 2 Institute for Stroke and Dementia Research (ISD), University Medical Center Munich, 81377 Munich, Germany; 3 Munich Cluster for Systems Neurology (SyNergy), Munich, Germany

In the mortality report of London in the year 1665, stroke was listed as a rare disease, overshadowed by the abundance of infectious causes of death. However, with the advancements in medical treatment and demographic changes, the prevalence of stroke has undergone a profound transition. Today, stroke is a significant global health concern and a leading cause of disability and mortality.

Stroke has traditionally been studied in the context of vascular dysfunction and brain injury. Recent scientific advances have revealed a significant involvement of the immune system in stroke pathology. The emerging research field of stroke-immunology continuously unravels insights into the complex interplay between the immune system and the central nervous system in the context of stroke. The collection of reviews in this Special Issue of Seminars in Immunopathology explores the intricate mechanisms and dynamic interactions between immune responses and the central nervous system during acute and long-term phases of stroke.

The initial articles discuss the systemic innate myeloid responses to acute ischemic and hemorrhagic stroke, shedding light on the roles of myeloid cells and their contributions to the immune response in stroke [1]. Additionally, the importance of sex differences in the inflammatory response to stroke highlights the need to consider sex-specific immune modulation in stroke research and treatment [2]. Distinct immune cell subsets contribute differently to stroke pathology, as discussed with regard to the functions of B cells and their impact on stroke outcomes, providing new insights for potential therapeutic interventions targeting these immune cells [3]. Along this line, the therapeutic potential of regulatory T lymphocytes as a therapy for ischemic stroke, aiming to understand the immunomodulatory strategies that may promote neuroprotection and functional recovery in stroke patients is also explored [4]. Next, the role of the ATP-adenosine axis to activate immune cells in ischemic stroke is discussed, revealing the molecular signaling pathways that influence immune responses and tissue damage in the ischemic brain. This review offers potential avenues for therapeutic targeting based on the modulation of ATP and adenosine signaling [5]. Considering the influence of age on immune responses, post-stroke innate immune responses between younger and older individuals are compared, unraveling age-related immunological changes that affect stroke outcome. Hence, this article underscores the importance of personalized approaches to stroke management [6]. The role of glia and the extracellular matrix in controlling neuroplasticity in the central nervous system is examined, shedding light on the interactions between immune components and neural regeneration following stroke, hereby providing insights into potential therapeutic strategies for stroke recovery [7]. Thromboinflammatory challenges in stroke pathophysiology are also highlighted, outlining the interplay between thrombosis and inflammation and their contributions to stroke progression and complications [8]. Additionally, the role of circulating cell-free DNA as an inflammatory mediator after stroke is explored, considering its potential as a biomarker and therapeutic target [9]. The importance of alarmins in post-stroke inflammation and neuronal repair is investigated, uncovering their impact on stroke outcomes and potential therapeutic applications [10]. Lastly, immune cell trafficking at the brain border zones in health and neurovascular diseases is reviewed, providing insights into the immune dynamics within the brain [11]. These articles highlight the critical role of brain-immune interactions in maintaining homeostasis and their potential dysregulation in neurovascular disorders.

Overall, this comprehensive collection of reviews offers an exploration of the immunopathology of stroke, synthesizing diverse perspectives to unravel the intricate immune responses and their impact on stroke pathophysiology. By shedding light on the complex interplay between the immune system and the central nervous system in stroke, these articles aim to stimulate further research, foster collaboration, and drive the development of novel diagnostic tools and targeted therapies.
